# Peripheral Disciform Degeneration: A Diagnostic Challenge

**DOI:** 10.7759/cureus.87175

**Published:** 2025-07-02

**Authors:** Anshul Radotra, Mehar Chawla, Sabitha Bandi, Nonavinakere Manjunatha

**Affiliations:** 1 Ophthalmology, King’s College Hospital National Health Service (NHS) Foundation Trust, London, GBR; 2 General Practice, Imperial College London, London, GBR; 3 Ophthalmology, University Hospital Coventry, Coventry, GBR

**Keywords:** chorioretinopathy, ocular oncology & medical retina, ophthalmology, painless vision loss, progressive vision loss

## Abstract

Peripheral disciform degeneration is a rare condition that presents significant diagnostic challenges due to its varied nomenclature and ability to mimic other ocular pathologies, such as choroidal melanoma. An 81-year-old female with longstanding poor vision in her right eye experienced progressive visual deterioration in her only functioning left eye. She underwent urgent evaluation in the eye casualty, followed by further assessment by the medical retina and ocular oncology teams to rule out the possibility of malignancy. Clinical examination and imaging revealed sub-foveal fluid and a hemorrhagic pigment epithelial detachment in the superotemporal quadrant, with angiography demonstrating blocked fluorescence. Based on these findings, a diagnosis of peripheral disciform degeneration was established. Given the presence of sub-foveal involvement, treatment with intravitreal bevacizumab was initiated. A one-month follow-up revealed complete resolution of sub-foveal fluid and an improvement in vision to baseline. This case highlights the importance of increasing awareness of this condition to ensure accurate diagnosis and guide appropriate management, particularly when malignancy is a concern.

## Introduction

Peripheral disciform degeneration is an uncommon degenerative condition, synonymous with peripheral exudative haemorrhagic chorioretinopathy (PEHCR), eccentric disciform degeneration, and peripheral haemorrhagic detachment of the retinal pigment epithelium [1-3]. It describes a clinical entity occurring in the peripheral retina, similar to exudative age-related macular degeneration, affecting elderly patients [4]. A low incidence of peripheral disciform degeneration can present diagnostic difficulty, and thus the objective of this case report is to increase awareness of this condition and its key characteristic features for accurate diagnosis and prompt treatment [5]. In this report, we describe the unique presentation, evolution, and treatment of peripheral disciform degeneration in an 81-year-old female with deteriorating vision.

## Case presentation

An 81-year-old Caucasian woman, with a history of previous left branch retinal vein occlusion (BRVO), bilateral dry age-related macular degeneration (AMD), and right eye blunt trauma, was routinely, virtually followed up in the medical retina clinic. Upon telephone review, the patient reported feeling very worried, as in her only functioning eye (left eye), she had noted worsening vision, a peripheral shadow, floaters, and flashing lights over the past three months.

**Figure 1:**
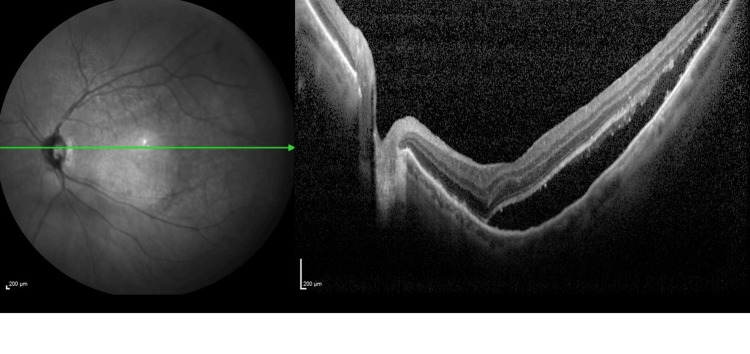
OCT image 1 of the left eye The image is showing subretinal fluid at the macula at presentation. OCT: Optical coherence tomography

**Figure 2:**
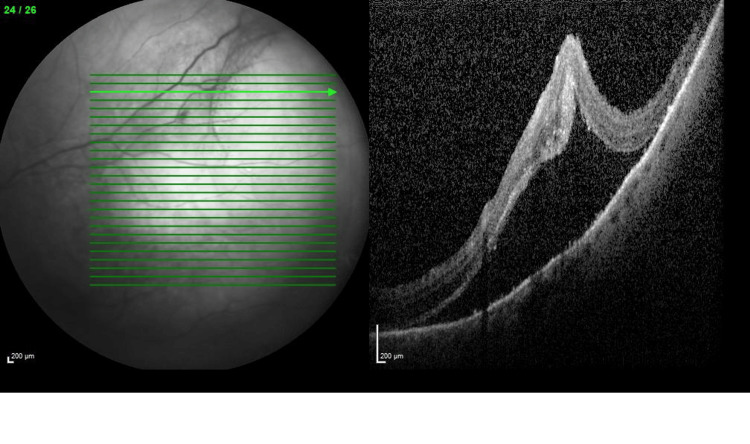
OCT image 2 of the left eye The image is showing peripheral subretinal fluid and retinal traction at presentation. OCT: Optical coherence tomography.

She was booked into the eye casualty as an emergency appointment, and on initial assessment, she had no perception of light (NPL) vision in her right eye and logMAR 1.0 (6/60) vision in her left. Baseline vision from 11 months prior was: right perception of light, left 0.9 (6/48). The anterior segments on examination were quiet, with the presence of posterior chamber intraocular lenses in both eyes. In addition, in the left eye, there was evidence of posterior capsular fibrosis and peripheral lens remnants. On fundal examination and optical coherence tomography (OCT), the right disc was pale, and in the left eye, there was the presence of subretinal fluid at the macula and retinal traction (Figures 1, 2). The patient was subsequently booked for review in the medical retina clinic in one week's time with fundus fluorescein angiography (FFA) and indocyanine green angiography (ICGA) to exclude occult choroidal neovascularisation (CNV).

Review in the medical retina clinic revealed supero-temporal retinal elevation and subretinal fluid on left fundal examination. Differential diagnoses of peripheral CNV, traction related to previous BRVO, and tumour were considered. A Vitreoretinal opinion was also sought, but no obvious retinal detachment or tumour was seen. FFA and ICGA evaluation did not reveal CNV; however, it demonstrated evidence of blocked fluorescence. A repeat OCT showed a shift in subretinal fluid compared to the previous imaging. In light of the above, the patient was referred to The Royal Liverpool University Hospital, Department of Ocular Oncology, to rule out the possibility of a tumour.

In Liverpool, repeat imaging, investigation, and examination were conducted as per their standard protocol. Left eye sub-foveal fluid was found, with presence of a haemorrhagic pigment epithelial detachment (PED) in the supero-temporal quadrant measuring 4.24 x 3.74 mm with a thickness of 0.94 mm on ultrasound, and infero-temporal exudation was also noted. Subsequently, when considering all clinical signs in unity, the patient was diagnosed with peripheral disciform degeneration. Typically, in most cases of peripheral disciform degeneration, no central involvement is present; however, in our patient, this was not the case. Targeted approaches such as cryotherapy and laser were considered initially; however, due to the impaired view of the peripheral fundus, these were not possible. Due to the presence of sub-foveal fluid, intravitreal injection of anti-vascular endothelial growth factor (Anti-VEGF) treatment bevacizumab was started, as per local protocol.

**Figure 3:**
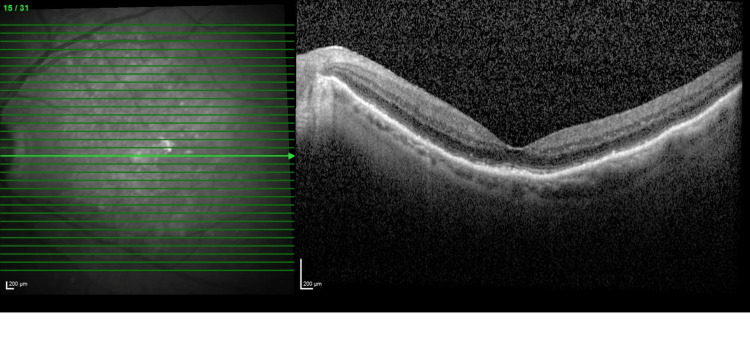
OCT image of the left eye showing a dry macula one month following bevacizumab treatment OCT: Optical coherence tomography.

**Figure 4:**
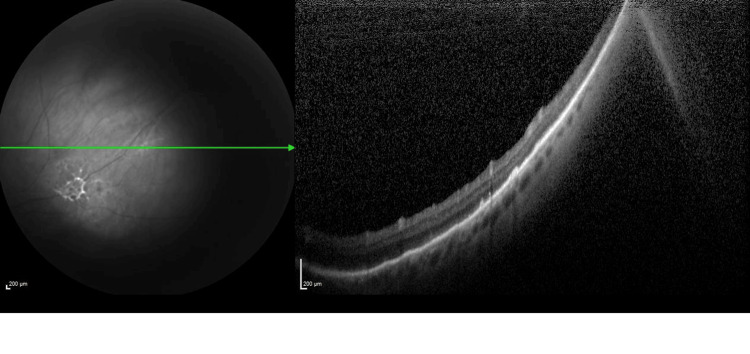
OCT image of the left eye at one month following bevacizumab treatment The image is showing the resolution of the peripheral subretinal fluid after one month of treatment. OCT: Optical coherence tomography.

A one-month follow-up post the first injection of bevacizumab showed anatomical improvement with regression and resolution of the sub-foveal fluid (Figures 3, 4) and functional improvement with vision returning to baseline: right eye (RE) NPL, left eye (LE) 0.9 (6/48).

**Figure 5:**
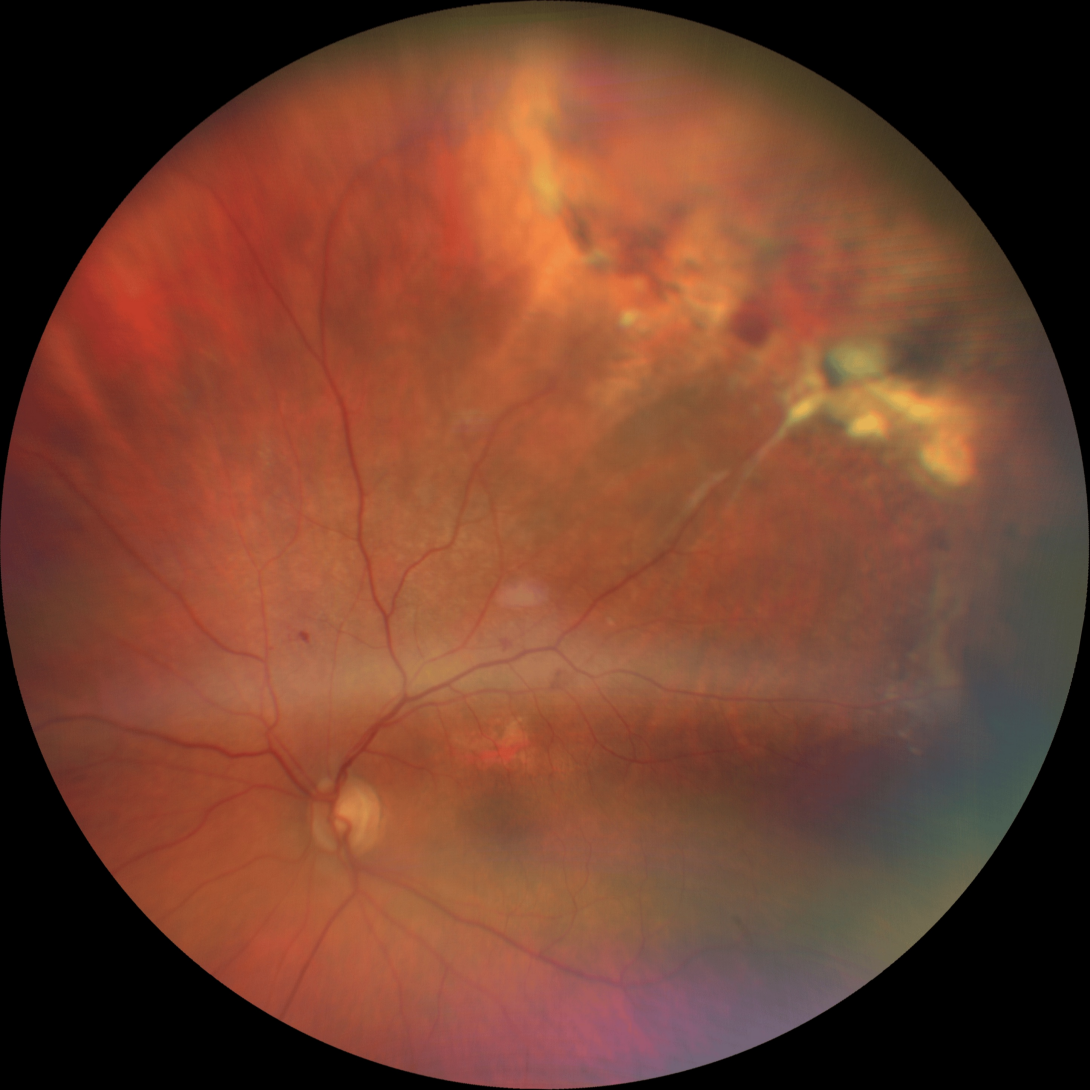
Wide field colour fundal image of the left eye post-treatment The image is showing supero-temporal peripheral disciform degeneration and retinal traction.

**Figure 6:**
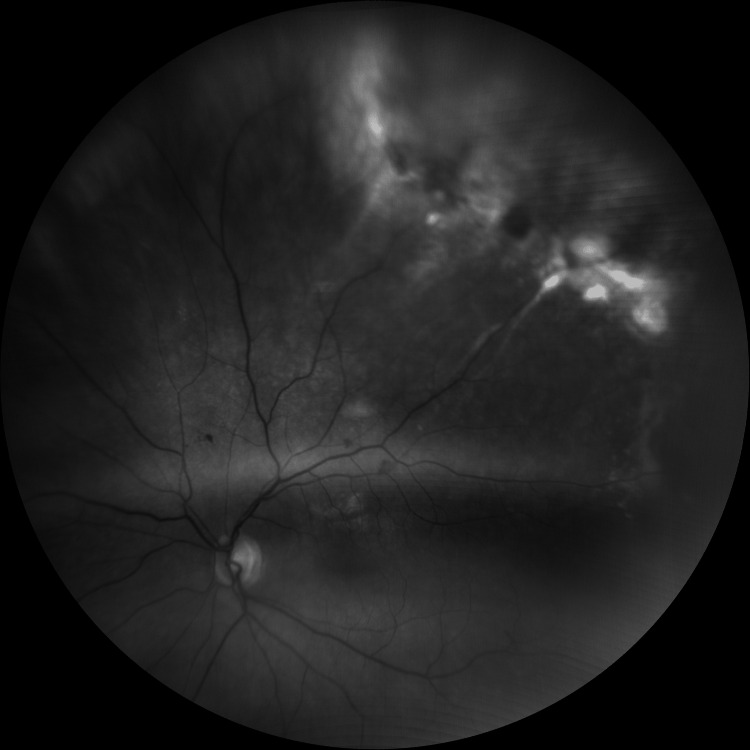
Wide field red-free fundal image of the left eye post-treatment The image is showing supero-temporal peripheral disciform degeneration.

Wide-field fundal images (Figures 5, 6) show representative images of the responsible supero-temporal peripheral disciform degeneration and traction post-treatment.

## Discussion

Peripheral disciform degeneration is a clinical entity similar to exudative age-related macular degeneration, occurring in the mid-peripheral and peripheral retina [1,6]. It is a rare condition, which predominantly affects elderly Caucasian women, with a mean age ranging from 70-82 years, with bilateral lesions present in 18-37% of cases [1,2,7,8]. It manifests as either isolated or multiple haemorrhagic or exudative lesions that consist of a PED with haemorrhage, sub-retinal fluid, and/or fibrotic scarring [8]. Hypertension, use of anti-coagulants, and history of age-related macular degeneration are known risk factors [2,7]. The exact aetiology of this condition is unknown; however, defects in Bruch’s membrane, ischaemia, mechanical forces, and peripheral neovascularisation have all been postulated [8,9].

Peripheral disciform degeneration poses a diagnostic challenge due to variations in nomenclature, rare occurrence, and lack of awareness and knowledge regarding the condition. In addition, it is known to mimic many other conditions. The differential diagnoses include: retinal capillary haemangioma, macroaneurysm, vasoproliferative tumour, familial exudative vitreoretinopathy, choroidal haemangioma, uveal lymphoma, choroidal metastatic tumour, and most importantly, choroidal melanoma [1,4,7]. The accurate and timely diagnosis is extremely important as patients can be saved from unnecessary radiation, enucleation, or other life-changing treatments [8,10]. These diagnostic difficulties were encountered in the indexed case above, and hence the patient was referred to Liverpool for further assessment in order to rule out malignancy.

Peripheral disciform degeneration and choroidal melanoma can both appear as elevated dark masses in the peripheral retina and share blockage of choroidal fluorescence on FFA; however, the pathophysiology is different in both [1,7]. Whilst in peripheral disciform degeneration, blockage of choroidal circulation is due to the presence of subretinal fluid and/or haemorrhage, in melanoma, this sign is usually seen secondary to RPE proliferation [5,7]. Choroidal melanoma will also show intrinsic vascularity with double circulation on FFA, which isn’t present in peripheral disciform degeneration. B scans are a useful tool in distinguishing the two entities. Both demonstrate elevated, dome-shaped masses; however, unlike choroidal melanoma, no choroidal excavation is seen in peripheral disciform degeneration [1,7]. Table 1 summarizes the different features [1,7,9,10].

**Table 1:** Characteristics of peripheral disciform degeneration and choroidal melanoma RPE: Retinal pigment epithelial, FFA: Fundus fluorescein angiography.

	Peripheral disciform degeneration	Choroidal melanoma
Demographics	Female predominance, average age 70-82 years	Male predominance, average age 59-62 years
Laterality	Unilateral in 67% Bilateral in 33%	Unilateral in >99%
Fundus features	Solitary or multiple peripheral retinal lesions, most commonly temporally, subretinal haemorrhage Sub-RPE haemorrhage, RPE detachment or tear, subretinal fibrosis, lipid exudation, vitreous haemorrhage	Solitary pigmented mass, located from the macula to equator, dark appearing mass in the majority of cases, with overlying orange pigment, exudative retinal detachments, Drusen rarely haemorrhagic
FFA findings	Blocked choroidal fluorescence by haemorrhage, peripheral hyperfluorescence due to RPE atrophy, negative double circulation pattern	Blockage effect due to choroidal mass, positive double circulation pattern, early hyperfluorescence with late staining and leakage, hot spots: multiple pinpoint subretinal hyperfluorescence
Ultrasound	Presence of retraction cleft and dome or plateau-shaped mass, absence of vascular pulsation, choroidal excavation, and shadowing of the orbit, variable internal reflectivity	Mushroom or dome-shaped mass, presence of choroidal excavation, orbital shadowing, and vascular pulsation, low to medium internal reflectivity

Peripheral disciform degeneration is usually a self-limiting condition that only requires close observation; however, in certain cases, as mentioned above, where vision is threatened, treatment options considered are laser photocoagulation, cryotherapy, photodynamic therapy (PDT), and intravitreal injection of Anti-VEGF [1,8]. Anti-VEGF injections of bevacizumab prevent the growth of and further damage from abnormal blood vessels and have previously been shown to provide good visual improvement and resolution of pre-existing subretinal hemorrhages, whilst stabilizing lesion size [11,12].

## Conclusions

In conclusion, this case report describes a case of an 81-year-old Caucasian female with peripheral disciform degeneration affecting central vision. It is a rare condition that provides diagnostic challenges as it mimics both benign and malignant conditions. This article highlights the importance of raising awareness of this condition for correct diagnosis and management, and avoidance of unnecessary interventions. Future research may focus on developing standardized diagnostic criteria, exploring long-term outcomes of Anti-VEGF therapy, and evaluating alternative treatment strategies for this condition.
